# Intratumoral high endothelial venules in solid tumors: a pooled study

**DOI:** 10.3389/fimmu.2024.1401118

**Published:** 2024-07-08

**Authors:** Bin Wang, Yin Han, Jie Liu, Xinyao Zhang, Yaotiao Deng, Yu Jiang

**Affiliations:** ^1^ Medical Oncology, Cancer Center, West China Hospital, Sichuan University, Chengdu, China; ^2^ Cancer Prevention and Treatment Institute of Chengdu, Department of Pathology, Chengdu Fifth People’s Hospital (The Second Clinical Medical College, Affiliated Fifth People’s Hospital of Chengdu University of Traditional Chinese Medicine), Chengdu, China

**Keywords:** solid tumor, high endothelial venules, prognosis, clinicopathological parameters, immunotherapy

## Abstract

**Objective:**

We performed this pooled analysis for the first time to comprehensively explore the prognostic value of tumor-associated high endothelial venules (TA-HEVs) and determine their relationships with clinicopathological features in solid tumors.

**Methods:**

Four online databases, including PubMed, Web of Science, Embase, and Cochrane Library, were comprehensively searched to identify studies assessing the effect of TA-HEVs on prognosis or clinicopathological features. Hazard ratios (HRs) with 95% confidence intervals (CIs) were applied to evaluate survival outcomes, including overall survival (OS), disease-free survival (DFS), progression-free survival (PFS), and cancer-specific survival (CSS). The association between TA-HEV status and clinicopathological characteristics was assessed by odds ratios (ORs) combined with 95% CIs. Subgroup analysis was conducted to explore sources of heterogeneity. The sensitivity analysis was performed to evaluate the stability of our findings. Meanwhile, Funnel plots were employed to visually evaluate potential publication bias, and both Begg’s and Egger’s tests were adopted to quantitatively determine publication bias.

**Results:**

A total of 13 retrospective cohort studies, involving 1,933 patients were finally included in this meta-analysis. Effect-size pooling analysis showed that the positivity of TA-HEVs was related to improved OS (pooled HR: 0.75, 95% CI: 0.62-0.93, *P*<0.01), and DFS (pooled HR = 0.54, 95% CI = 0.41-0.72, *P*< 0.01). However, TA-HEV positivity in solid tumors was not linked to PFS (pooled HR = 0.75, 95% CI 0.34-1.64, *P* = 0.47) or CSS (pooled HR: 0.58, 95% CI: 0.04-7.58, P= 0.68). Further subgroup analysis demonstrated that ethnicity and source of HR were the main factors contributing to heterogeneity. Moreover, TA-HEVs were inversely associated with lymph node metastasis and distant metastasis, but were positively related to worse tumor differentiation. However, TA-HEVs were not significantly correlated with sex, LVI, clinical stage, and depth of invasion. Sensitivity analysis suggested that the pooled results were stable and reliable, with no significant publication bias in all included articles.

**Conclusions:**

This is the first comprehensive analysis of the prognostic value of TA-HEVs in solid tumors using existing literature. Overall, our study demonstrated a significant correlation between TA-HEVs and prognosis as well as clinicopathological features. TA-HEVs may serve as novel immune-related biomarkers for clinical assessments and prognosis prediction in solid tumors.

**Systematic review registration:**

https://www.crd.york.ac.uk/prospero/display_record.php, identifier CRD42023394998.

## 1 Introduction

Cancer immunotherapies based on T cells through the restoration of host anti-tumor immune responses have witnessed unprecedented advances in the last decade, which has transformed the treatment paradigm for multiple tumor types (1). Despite impressive therapeutic efficacy in subsets of patients, most patients exhibit innate or acquired resistance to these therapies (1). Mounting clinical evidence has indicated that abundant infiltration of malignant lesions by immune effector cells is a prerequisite for recognizing and killing tumor cells (2). However, these immune effector cells are largely excluded from the tumor microenvironment (TME), partly caused by abnormal alterations of the angiogenic vasculature, which highlights the essential role of tumor vasculature in anti-tumor immunity and immunotherapies (3).

A particular form of vasculature is high endothelial venules (HEVs) which are specialized and organ-specific postcapillary venules (4). HEVs express high levels of peripheral node addressing (PNAd) that are identified by the HEV-specific antibody MECA-79, which is uniquely poised to mediate the capture and rolling of lymphocytes towards secondary lymphoid organs like lymph nodes (LNs) (4). The HEV network expands during inflammation in immune-stimulated LNs and is profoundly remodeled in metastatic and tumor-draining LNs, facilitating the amplification and maintenance of chronic inflammation (5). HEVs can also form ectopically in a wide variety of cancers, usually surrounded by dense infiltration of lymphocytes organized into lymph-node-like, T- and B-cell-rich areas referred to as tertiary lymphoid structures (TLSs) (6). Multiple studies have demonstrated a close correlation between HEV density and the density of TLSs (7, 8). As a special component of TLSs, tumor-associated HEVs (TA-HEVs) serve as gateways for the recruitment of lymphocytes to TLSs, and once recruited, naive lymphocytes are activated locally by tumor antigens (7). The formation of TA-HEVs may overcome the major barrier of immune effector cell exclusion from the TME, providing attractive avenues to induce and sustain the efficacy of immunotherapy (9). Many studies have found positive correlations between TA-HEVs and favorable prognosis in various types of cancer, such as non-small cell lung cancer, melanoma, breast cancer, and colorectal carcinoma (10). However, the opposite effect has been reported for TA-HEVs in many tumors like colorectal cancer (11) and gastric cancer (12). Thus, the clinical significance of TA-HEVs across various solid tumors remains controversial, which poses a major obstacle to their clinical application as biomarkers to accurately predict prognosis and immunotherapy response.

However, no meta-analysis has thus far been conducted to assess the role of TA-HEVs in solid tumors. To address this gap, we performed this pooled analysis for the first time to comprehensively explore the prognostic value of TA-HEVs and determine their relationships with clinicopathological features in solid tumors.

## 2 Methods

We performed this study in accordance with the Preferred Reporting Items for Systematic Reviews and Meta-analyses (PRISMA) guidelines (13). Additionally, this study has been registered in PROSPERO (registration number: CRD42023394998).

### 2.1 Search strategy

A comprehensive search of online databases, including PubMed, EMBASE, Web of Science, and the Cochrane Library, was conducted to identify eligible studies from their inception up to May 2023. The following terms and their combinations applied in our search: (‘High Endothelial Venules’ OR ‘HEVs’) AND (‘neoplasm’ OR ‘neoplasia’ OR ‘cancer’ OR ‘tumor’ OR ‘malignant neoplasm’ OR ‘malignancy’ OR ‘carcinoma’). In addition, the references of the retrieved articles were manually searched for more eligible studies.

### 2.2 Inclusion and exclusion criteria

The published article considered as eligible must meet the following inclusion criteria: (1) involved patients must be histopathologically diagnosed with cancer; (2) the status of TA-HEVs were determined by immunohistochemistry (IHC) method with MECA-79 as HEV-specific marker; and (3) The relationship between TA-HEVs and or clinicopathological parameters was investigated; The exclusion criteria were listed as follows: (1) reviews, editorials, letters, case reports, conference abstracts, or unpublished articles; (2) studies without useable data for survival outcomes or clinicopathological parameters; (3) literature with overlapped data; and (4) non-English articles.

### 2.3 Data extraction

The data were extracted independently by two investigators utilizing a standardized data-extract form, and any disputes were settled by consensus involving a third investigator. We fetched the following information and data from each study: (a) basic characteristics: first author, publication year, country, cancer type, sample size, median age, follow-up time, detection method, detection marker, cut-off criteria, and study design; (b) clinicopathologic parameters: case number in negative-and positive-HEVs groups after stratification by sex, histological grade, lymphovascular invasion (LVI), clinical stage, invasion of depth, lymph node metastasis, and distant metastasis; (c) measures of prognosis: hazard ratios (HRs) and with corresponding 95% CIs for overall survival (OS), disease-free survival (DFS), progression-free survival (PFS), and/or cancer-specific survival (CSS). If both univariate and multivariate analyses were applied to calculate the HRs, the latter was preferred to avoid the influence of confounding factors. If survival outcomes were not available in the original studies, HRs, and 95%CIs were retrieved from Kaplan-Meier (K-M) curves using Engauge Digitizer software (version 4.1) and Tierney’s reported method (14).

### 2.4 Quality assessment

The quality of the included studies was assessed independently by two investigators according to the Newcastle–Ottawa Quality Assessment Scale (NOS) (15). Scores for quality assessment ranged from 0 to 9, with studies scoring 6 or higher considered high-quality.

### 2.5 Statistical analysis

All statistical analyses in this study were conducted by the R Software (version 4.1.1). Pooled HRs with 95% CIs were applied to evaluate the relationship between positive-HEVs and survival outcomes. The correlations between positive-HEVs and clinicopathological features were assessed by pooled odds ratios (ORs) with 95% CIs.*I^2^* and Cochran’s Q statistics were applied to assess the heterogeneity among these articles. When the heterogeneity was statistically significant (*I^2^* > 50% and *P* > 0.10), the random effect model was chosen; otherwise, the fixed effect model was conducted. The source heterogeneity and stability of results were conducted by subgroup analysis and sensitivity analysis, respectively. Meanwhile, Funnel plots were employed to visually evaluate potential publication bias, and both Begg’s and Egger’s tests were adopted to quantitatively determine publication bias. A *P*-value of less than 0.05 implied statistical significance.

## 3 Results

### 3.1 Study identification

**Figure 1:**
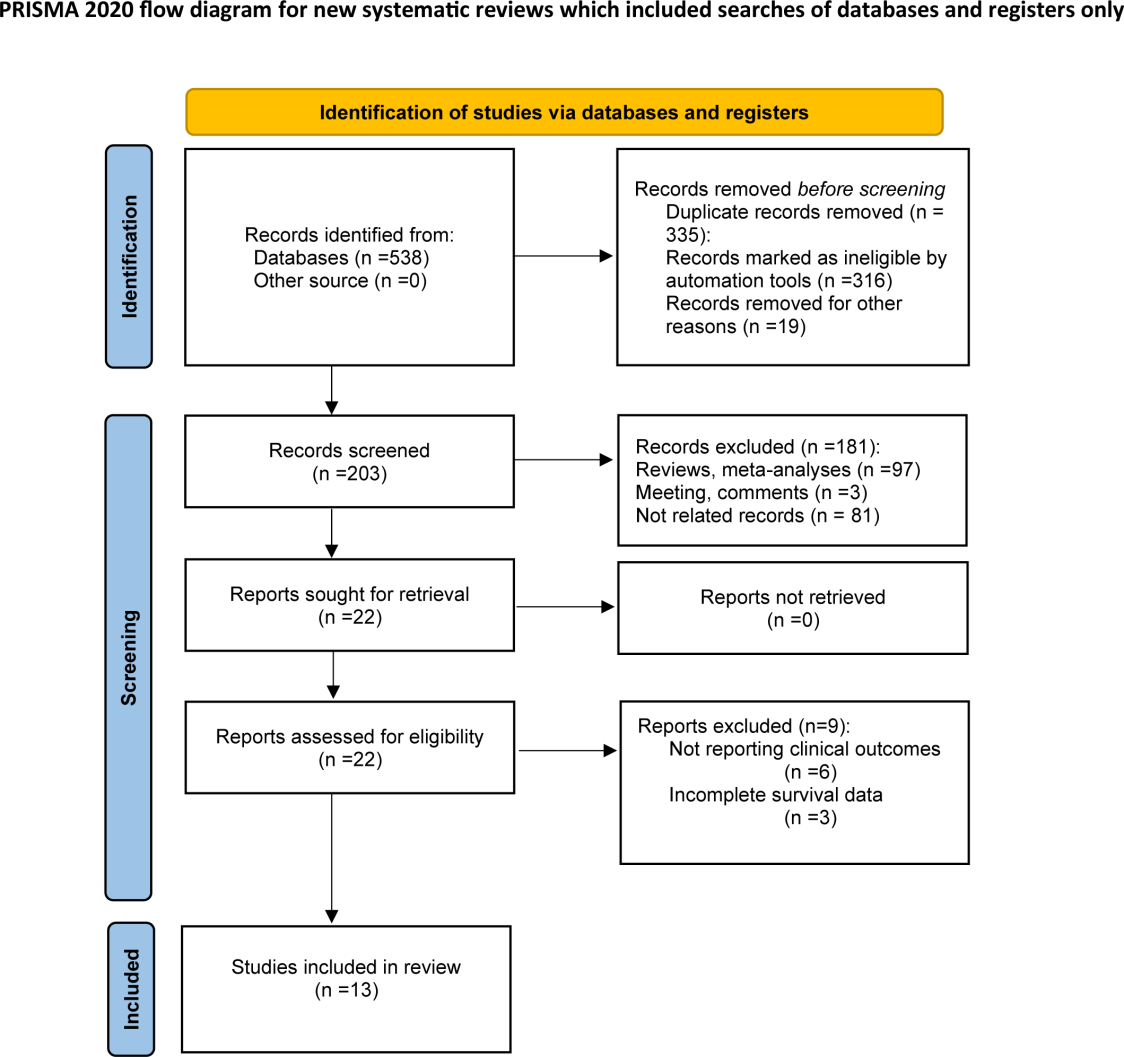
Flow diagram of the study selection process.

According to the above retrieval strategy, a total of 538 records were identified from databases and manually searched. After the removal of 335 duplicates, 203 studies were retained for subsequent examination. Through abstract and title screening, 181 articles were excluded because of meta-analysis, reviews, comments, and non-relevance with the theme. After a full‐text assessment of the remaining 22 potentially eligible records in detail, 9 were excluded for not meeting eligibility criteria. Finally, thirteen articles comprising 1,933 patients were included in this study for further analysis. The selection process is illustrated in **Figure 1**.

### 3.2 Study characteristics

**Table 1** listed the basic characteristics of the qualified articles included in this meta-analysis. All studies were performed retrospectively between 2011 and 2023, with a sample size ranging from 40 to 452. Geographically, these studies were conducted in six countries (4 in Korea, 4 in France, 2 in China, 1 in Hungary, 1 in Japan, and 1 in Finland). The types of cancers in the enrolled studies were gastric cancer (12, 19, 21), head and neck squamous cell carcinoma (HNSCC) (16, 20, 26), breast cancer (10, 17), melanoma (18, 23), esophageal squamous cell carcinoma (ESCC) (22), colorectal cancer (25), and endometrial cancer (24), respectively. Nine of the 13 included studies investigated the correlation between clinicopathological parameters and TA-HEVs (sex, 6 studies; histological grade, 4 studies; LVI, 3 studies; clinical stage, 3 studies; invasion of depth, 6 studies; lymph node status, 6 studies; distant metastasis status, 3 studies);. Twelve of the 13 enrolled studies evaluated prognostic values of TA-HEVs, with 8 assessing OS, 5 assessing DFS, 2 assessing PFS, and 2 assessing CSS. As for quality assessment, all included studies with a NOS score of 6 or higher suggested that the quality of the included studies was relatively high (**Supplementary Table S1**).

**Table 1:** Main characteristics of the eligible studies.

Eligible Study	Year	Country	Caner type	Sample size	Median age (range)	Follow-up time (months)	Assay method	Therapy	HEV marker	Cutoff criteria	Survival outcome	Source of HR	Study design
Martinet L et al. (10)	2011	France	Breast cancer	146	NR	122	IHC	Surgery	MECA-79	The highest tercile vs. 2 lowest terciles	OS; DFS	Reported	Retrospective
Okayama H et al. (12)	2011	Japan	Gastric cancer	250	NR	83 (1.7–213.6)	IHC	Surgery	MECA-79	More than 5%	CSS	Reported	Retrospective
Wirsing AM et al. (16)	2016	Korea	OSCC	108	42(23- 70)	NR	IHC	Surgery	MECA-79	Median value	CSS	Reported	Retrospective
Song IH et al. (17)	2017	Korea	TNBC	108	42(23-70)	31.4(21.1-53.0)	IHC	NAC and surgery	MECA-79	NR	DFS	Reported	Retrospective
Sebestyén T et al. (18)	2018	Hungary	Melanoma	118	NR	NR	IHC	Surgery	MECA-79	Median value	DFS; OS	Survival curve	Retrospective
Hong SA et al. (19)	2020	Korea	Gastric Cancer	157	66.3 (35–92)	43	IHC	Surgery	MECA-79	Present vs. absent	OS	Reported	Retrospective
Karpathiou G et al. (20)	2021	France	HNSCC	135	62.2 (40-86)	NR	IHC	NAC or surgery	MECA-79	Mean value	OS; PFS	Survival curve	Retrospective
Park HS et al. (21)	2021	Korea	Gastric cancer	452	NR	NR	IHC	Surgery	MECA-79	Median value	OS; RFS	Reported	Retrospective
Li H et al. (22)	2022	China	ESCC	52	59(44-76)	NR	IHC	Surgery	MECA-79	Median score	OS	Survival curve	Retrospective
Asrir A et al. (23)	2022	France	Melanoma	93	NR	NR	IHC	Anti-PD-1 combined with anti-CTLA-4 Thepapy	MECA-79	TA-HEV scores 3 or 2	OS; PFS	Survival curve	Retrospective
Karpathiou G et al. (24)	2022	France	Endometrial cancer	40	62.5 (33–90)	24 (24-168)	IHC	Surgery	MECA-79	Present vs. absent	NR	NR	Retrospective
Zhan Z et al. (25)	2023	China	Colorectal cancer	203	NR	50 (0–72)	IHC	Surgery	MECA-79	Mean value	OS; DFS	Reported	Retrospective
Hyytiäinen A et al. (26)	2023	Finland	OSCC	71	61	39 (0 –97)	IHC	Surgery adjuvant RT or CRT	MECA-79	Median value	DFS	Survival curve	Retrospective

TNBC, triple-negative breast cancer; ESCC, esophageal squamous cell carcinoma; OSCC, oral tongue squamous cell carcinoma; OS, overall survival; DFS, disease-free survival; PFS, progression-free survival; CSS, cancer-specific survival; NR, not reported; IHC, immunohistochemistry; NAC, neoadjuvant chemotherapy; PD-1, Programmed death-1; CTLA-4, cytotoxic T lymphocyte–associated protein 4; RT, radiotherapy; CRT, chemoradiotherapy.

### 3.3 The prognostic value of TA-HEVs for OS

**Figure 2:**
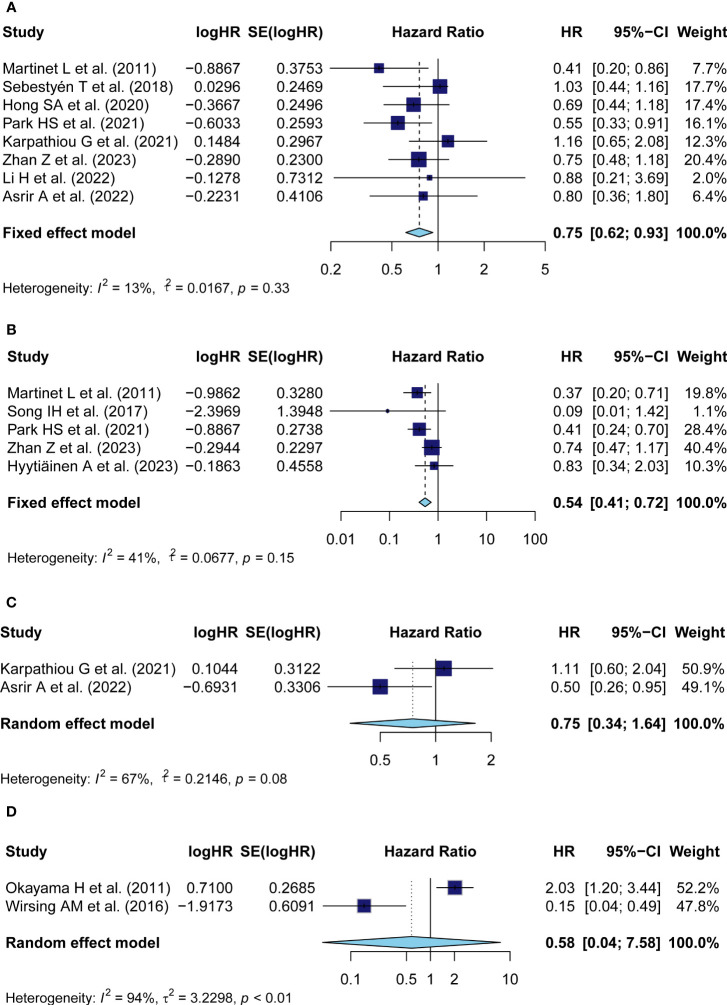
Meta-analysis of the prognostic value of TA-HEV positivity in solid tumors. **(A)** Forest plots for the association of HEV positivity with OS; **(B)** Forest plots for the association of TA-HEV positivity with DFS; **(C)** Forest plots for the association of TA-HEV positivity with PFS; **(D)** Forest plots for the association of HEV positivity with CSS. The HR <1 revealed that TA-HEV positivity was related to a favorable prognosis. Diamonds indicated overall HR with the corresponding 95% CI.

Eight studies with a number of 1,333 patients provided data on OS in relation to TA-HEV status. Since weak heterogeneity existed among articles (*I^2^* = 13%, *P* = 0.33), the fixed model was employed to calculate the pooled HR with 95% CI of OS. The combined result indicated that patients with positive-HEVs tended to be related to a favorable OS (pooled HR: 0.75, 95% CI: 0.62-0.93, *P*<0.01) (**Figure 2A**).

### 3.4 The prognostic value of TA-HEVs for DFS

The HRs in DFS were available in five studies comprising 754 patients. Similar to OS, HEV positivity was strongly interrelated with a better DFS (pooled HR = 0.54, 95% CI: 0.41-0.72, *P*< 0.01) (**Figure 2B**). A fixed effect model was performed as no significant heterogeneity was observed (*I^2^* = 41%, *P* = 0.15).

### 3.5 The prognostic value of TA-HEVs for PFS and CSS

Two studies reported on the prognostic effect of TA-HEVs on PFS. The pooled analysis with a random effect model demonstrated that HEV positivity in solid tumors was not linked to a better PFS (pooled HR = 0.75, 95% CI: 0.34-1.64, *P* = 0.47), with significant heterogeneity among studies (*I^2^* = 67%, *P* = 0.08) (**Figure 2C**); Data for CSS analysis was extracted from two studies comprising 385 patients. Consistently, because of significant heterogeneity (*I^2^* = 94%, P< 0.01), the HR was pooled employing the random-effect model and indicated that positive-HEVs were not significantly associated with CSS (pooled HR: 0.58, 95% CI: 0.04-7.58, P= 0.68) (**Figure 2D**).

### 3.6 Association between TA-HEVs and clinicopathological characteristics

**Figure 3:**
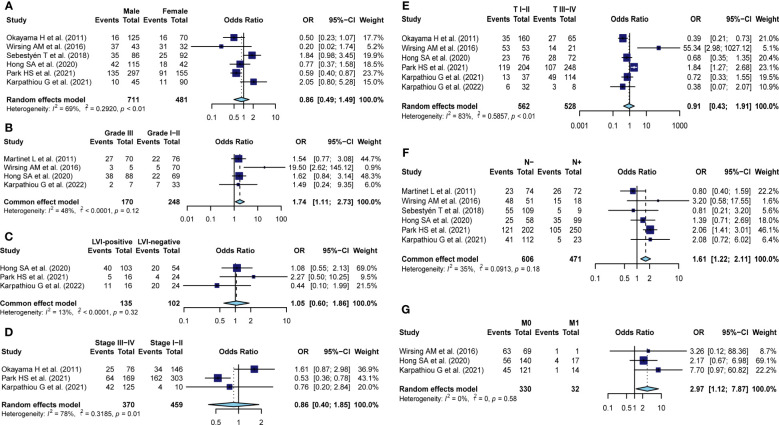
Meta-analysis for the association of TA-HEVs with clinicopathological parameters. Forest plots showed the correlation between HEV positivity and **(A)** sex (male vs. female), **(B)** Histological grade (III vs. I-II), **(C)** LVI status (positive vs. negative), **(D)** clinical stage (III-IV vs. I-II), **(E)** invasion of depth (I-II vs. III-IV), **(F)** nodal metastasis (no vs. yes), **(G)** distant metastasis (no vs. yes); Each result was shown by the OR with 95% CI. Diamonds indicated pooled OR with the corresponding 95% CI.

To comprehensively explore the clinical value of TA-HEVs in solid tumors, we investigated the correlation between TA-HEV status and multiple clinicopathological parameters from eleven studies, including sex, histological grade, LVI, clinical stage, invasion of depth, lymph node metastasis, as well as distant metastasis (**Figure 3**; **Supplementary Table S2**). The results of the pooled analysis demonstrated that TA-HEV positivity was related to negative lymph node metastasis (pooled OR = 1.61, 95% CI: 1.22-2.11, *P*< 0.01; *I^2^* = 34.7% *P*=0.18), and negative distant metastasis (pooled OR: 2.97, 95% CI: 1.27-7.87, *P*= 0.01; *I^2^* = 0%, *P*= 0.58), whereas a positive relation was found between TA-HEV positivity and worse histological grade (pooled OR: 1.74, 95% CI: 1.11- 2.73, *P*= 0.01; *I^2^* = 48%, *P*= 0.12). However, no significant associations were observed between TA-HEV positivity and sex (pooled OR: 0.86, 95% CI: 0.50-1.49, p=0.58; *I^2^* = 69%, *P*< 0.01), LVI (pooled OR: 1.05, 95% CI: 0.60-1.86 *P*= 0.85; *I^2^* = 13%, *P*= 0.32), clinical stage (pooled OR: 0.86, 95%CI: 0.40-1.85, *P*< 0.01; *I^2^* = 78%, *P*= 0.01), as well as invasion of depth (pooled OR: 0.91, 95% CI: 0.43-1.91, *P*= 0.80; *I^2^* = 83%, *P*< 0.01).

### 3.7 Subgroup analyses

To better detect the predictive prognostic effect of TA-HEVs, the subgroup analysis was conducted for OS, which was stratified by ethnicity, sample size, tumor type, cut-off criteria, and source of HR (**Table 2**; **Supplementary Figure S1**). The results indicated that HEV positivity showed significant prognostic value in Asians, but not in Caucasians. Subsequently, we found that HEV positivity in groups with sample size ≥ 200, directly reported HR and cut-off criteria with non-median values were more prone to be correlated with better OS. Further subgroup analysis of tumor type revealed that TA-HEVs were associated with the prognosis of patients with gastrointestinal cancer, whereas not related to others. Due to the limited number of included articles, more studies were required to investigate the relationships between TA-HEVs and different tumor types. We also found the absence of heterogeneity in some subgroups (*I^2^* = 0), including ethnicity, sample size (≥200), tumor type (gastrointestinal cancer), and source of HR.

**Table 2:** Subgroup analysis of the prognostic value of TA-HEVs for OS in solid tumor.

Subgroup analysis	No.of studies	Effect model	Pooled HR (95%CI)	Heterogeneity I^2^ (%)		*P*
OS						
Total	8	Fixed	0.75 (0.62, 0.93)	13	0.33	
Ethnicity						
Asian	5	fixed	0.63 (0.49, 0.82)	0	0.65	
Caucasian	3	fixed	1.03 (0.73, 1.44)	0	0.76	
Sample size						
<200	6	fixed	0.82 (0.64, 1.06)	17	0.30	
≥200	2	fixed	0.65 (0.47, 0.91)	0	0.36	
Tumor type						
Gastrointestinal cancer	4	Fixed	0.67 (0.51, 0.88)	0	0.8	
Others	4	Fixed	0.87 (0.64, 1.19)	45	0.14	
Cut-off value						
Median	3	Fixed	0.77(0.55, 1.08)	37	0.30	
Non-median	5	Fixed	0.75(0.58,0.96)	17	0.21	
Source of HR						
Reported	4	fixed	0.63 (0.48, 0.81)	0	0.51	
K-M curves	4	fixed	1.02 (0.73, 1.41)	0	0.90	

HR, hazard ratio; CI, confidence; OS, overall survival; K-M, Kaplan-Meier.

### 3.8 Sensitivity analysis

**Figure 4:**
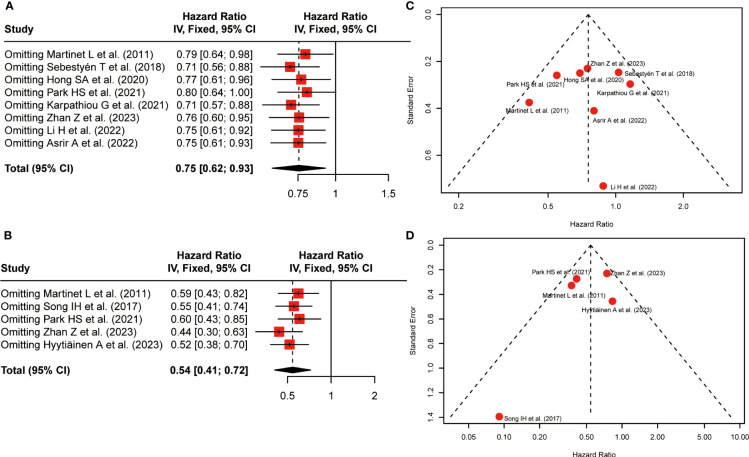
Sensitivity analysis and funnel plots. **(A)** Sensitivity analysis for the association between HEV positivity and OS; **(B)** Sensitivity analysis for the association between HEV positivity and DFS; **(C)** Funnel Plot to detect publication bias of positive-HEVs on OS; **(D)** Funnel Plot to detect publication bias of positive-HEVs and DFS.

To assess the stability of survival outcomes, a sensitivity analysis was employed by sequentially deleting a single study individually (**Figures 4A, B**). The final results showed that no individual study affected the pooled HR of OS and DFS, indicating that our results were stable and reliable.

### 3.9 Publication bias

Funnel plots were applied to qualitatively assess publication bias, and both Begg’s test and Egger’s test were performed to quantitatively calculate publication bias. Visual inspection of the funnel plot revealed no remarkable asymmetry (**Figures 4C, D**). *P-*values of Begg’s were 1.00 for OS and 0.22 for DFS, respectively. the *P*-values in Egger’s test were equal to 0.86 for OS and 0.43 for DFS, respectively. Thus, All *P*-values were more than 0.05, demonstrating less possibility of publication bias concerning OS and DFS.

## 4 Discussion

The lack of sufficient pre-existing intratumoral lymphocyte infiltration poses a major obstacle to effective immunotherapies, with tumor-infiltrating lymphocytes being strongly associated with improved clinical outcomes in various cancers (3). TA-HEVs may offer attractive avenues to initiate and sustain effective immunotherapies by overcoming immunocyte exclusion from the TME (8).TA-HEVs are considered to be of great importance in recruiting naive T cells and B cells into the tumors, and locally enhancing anti-tumor immunity by fostering the formation of TLSs (7). However, the prognostic significance of HEVs among various studies is still controversial. This study, for the first time, systematically investigated the prognostic values of TA-HEVs and determined their relationships with clinicopathological features in solid tumors.

To systematically elucidate the prognosis role of TA-HEVs in solid tumors, data were pooled from 13 studies involving 1,933 patients in the present study. The pooled analysis demonstrated TA-HEVs might be favorable prognostic biomarkers in solid tumors, with positive TA-HEVs being significantly correlated to better survival outcomes regarding OS and DFS. Moreover, sensitivity analysis and publication bias tests indicated that these results were stable and credible. Correspondingly, a positive correlation has been observed between TA-HEV density and tumor remission in patients with cutaneous melanoma (27). The presence of TLSs comprising TA-HEVs was reported to be an independent prognostic biomarker for pancreatic ductal adenocarcinoma patients treated with neoadjuvant chemotherapy (NAC), possibly resulting in a better prognosis (28). Song IH et al. (17) proposed that the high density of TA-HEVs not only predicted the pathologic complete response but was also a predictor of improved DFS in TNBC patients receiving NAC. A recent study conducted by Asrir A et al. (23) revealed that high numbers of TA-HEVs in pre-treatment metastatic lesions were associated with better clinical response and survival of melanoma patients undergoing combined immune checkpoint inhibitors (ICI) therapy. These findings are in line with the crucial role of HEVs as major portals for lymphocyte infiltration into tumors. Studies of multiple human malignancies have confirmed that lymphocyte infiltration specifically at HEV-rich areas of the tumors, and a strong correlation between HEV density and densities of tumor-infiltrating CD3^+^ T cells, CD8^+^ T cells, and CD20^+^ B cells (4, 10). TA-HEVs have been confirmed to be the main sites of lymphocyte tethering, rolling, and sticking in the tumor microcirculation both at baseline and during combined ICI therapy (23). Analysis of tumor biopsies from 93 metastatic melanoma patients revealed that high numbers of TA-HEVs were related to better survival and clinical response of patients treated with combined immunotherapy (23). A real-world retrospective study in advanced non-small cell lung cancer indicated that PNAd^+^ TA-HEVs were predictive of better response and survival upon PD-1 blockade combined with anti-angiogenesis therapy (29).

In the vast majority of our results, heterogeneity was not significant among the different studies, indicating that TA-HEVs have broad applicability across different populations and tumor types. Nonetheless, we still explored the potential source of heterogeneity based on PRISMA Guidelines. Subgroup analysis identified that ethnicity and source of HR might be potential sources of heterogeneity, with *P*-values of subgroup difference test less than 0.05. It was also found that the critical value of sample size (≥200), tumor type (others), and cut-off criteria with non-median values had no significant relationship with OS. This might be why the small sample size, limited studies, and non-uniform cut-off criteria could not reveal the real results. Notably, Recent studies suggested varying degrees and stages of TA-HEV maturation might imply functional differences among intertumoral MECA-79^+^ vessels. And, plump TA-HEVs surrounded by massive lymphocyte aggregates assumed to be more mature in comparison to some flat and isolated TA-HEVs located at the periphery (8). Moreover, it was worth noting that TA-HEV-related TLSs predominantly aroused at the tumor periphery or the tumor interphase, while HEVs in the tumor center were very rare and not correlated with well-organized immune cell aggregates. Besides, TA-HEVs could be present in DC- and T-cell-rich regions, as well as in B-cell-rich areas (6, 30). Wang et al. demonstrated that the rapid generation of synthetic human HEV-like structures by a tissue-bioengineering approach effectively enabled the formation of lymphoid structures with TLS functional properties *in vivo*, which acted as lymphatic hubs facilitating T cell infiltration into the TME and eliminate tumor cells (31). Mature HEVs, rather than lymphatics or blood vessels, have been shown to mediate CD8^+^ T cell infiltration, whereas the immune checkpoint ligands expressed on mature HEVs could negatively regulate CD8^+^ T cell entry into TLSs (32). There is currently still a debate to which extent TA-HEVs are necessary to actively influence cancer progression in TLSs or TLS-like structures (17). However, due to the lack of sufficient data, further assessment involving the impact of different maturation degrees and distributions of TA-HEVs on prognosis cannot be performed.

To comprehensively explore the possible factors affecting TA-HEV status in solid tumors, we assessed differences of common clinicopathological characteristics between the HEV-positive and HEV-negative patients. Our pooled results indicated that positive TAHEVs were inversely associated with nodal metastasis and distant metastasis. Similar to the current study, TA-HEVs were found to be negatively related to Clark level and Breslow thickness, and positively correlated with T-cell infiltration in primary melanomas (33). A higher density of TA-HEVs was observed in microsatellite-unstable colorectal carcinomas compared to microsatellite-stable tumors (25). Furthermore, HEV-high gastric tumors exhibited increased immune-modulating chemokines, activated pathway of type I or II interferon, and upregulated immune checkpoints such as PD-1 and TIGIT (21). These links might suggest that induction of TA-HEVs was most pronounced during the initial stages of tumor development when the immune response was expected to be the highest. Consequently, the presence of TA-HEVs might be a good proxy to assess the strength of ongoing anti-tumor immune responses. However, regarding sex, LVI, clinical stage, and depth of invasion, no significant difference was observed between HEV-positive and HEV-negative patients. Additionally, we discovered a positive correlation between TA-HEVs and worse tumor differentiation. One possible explanation was that poorly differentiated tumor cells tended to trigger early immune responses within the tumor, resulting in increased production of TA-HEVs, and facilitating immune cell infiltration into the TME (22). However, further studies are required to fully explore underlying mechanisms involved in this process.

Given these results, TA-HEV-inducing strategies may represent an exciting prospect for future cancer immunotherapy. Although our study revealed that TA-HEVs could act as valuable prognostic biomarkers in solid tumors, the results should be interpreted with caution. First, the cut-off values for TA-HEVs among the included studies were inconsistent, which might affect the evaluation of their clinical value. Second, the HRs and 95% CIs of some included articles were indirectly extracted from KM curves, which were less reliable than those directly obtained from original articles. Third, the number of included studies was limited, and all had small sample sizes and were retrospective. Moreover, treatment options varied significantly across different cancer types, which might influence the predictive value of TA-HEVs.

## 5 Conclusion

Overall, this is the first comprehensive analysis to explore the prognostic value of TA-HEVs and determine their relationships with clinicopathological features in solid tumors based on existing literature. Despite the aforementioned limitations, our results demonstrated that TA-HEVs might serve as new immune-related biomarkers for clinical assessments and prognosis prediction in solid tumors, providing clinical basses for cancer individualized treatment. However, further exploration with a larger sample size and a unified detection method is required to assess potential effects of TA-HEVs on different solid tumors.

## Data availability statement

The original contributions presented in the study are included in the article/**Supplementary Material**. Further inquiries can be directed to the corresponding author.

## Author contributions

BW: Conceptualization, Software, Validation, Writing – original draft, Writing – review & editing. YH: Formal analysis, Investigation, Methodology, Writing – review & editing. JL: Formal analysis, Funding acquisition, Writing – original draft. XZ: Resources, Visualization, Writing – review & editing. YD: Resources, Software, Writing – review & editing. YJ: Funding acquisition, Visualization, Writing – review & editing.
