# Supplementary material for: Intratumoral high endothelial venules in solid tumors: a pooled study
Source: Front Immunol. 2024 Jul 8;15:1401118. doi: 10.3389/fimmu.2024.1401118 (PMC11260642; doi:10.3389/fimmu.2024.1401118)
Supplement: Supplementary file 1 [file DataSheet_1.pdf]

Additional file

Table S1. Newcastle-Ottawa Scale for assessing the quality of studies in quantitative analysis

Study ID	Year	Representativeness of the exposed cohort	Selection of the non-exposed cohort	Ascertainment of exposure	Demonstration that outcome of interest was not present at start of study	Comparability of cohorts on the basis of the design or analysis (a)	Comparability of cohorts on the basis of the design or analysis (b)	Assessment of outcome	Was follow-up long enough for outcomes to occur	Adequacy of follow-up of cohorts	Total scores
Martinet L, *et al*	2011	★	★	★	★	★	★	★	★	★	9
Okayama H, *et al*	2011	★	★	★	★	★	★	★	☆	★	8
Song IH, *et al*	2017	★	★	★	★	★	☆	★	☆	★	7
Wirsing AM, *et al*	2018	★	★	★	★	★	☆	★	☆	★	7
Sebestyén T, *et al*	2018	★	★	★	★	★	☆	★	★	★	8
Hong SA, *et al*	2020	★	★	★	★	★	★	★	☆	★	8
Karpathiou G, *et al*	2021	★	★	★	★	☆	☆	★	★	★	7
Park HS, *et al*	2021	★	★	★	★	★	★	★	★	☆	8
Li H, *et al*	2022	★	★	★	★	★	★	★	★	☆	8
Asrir A, *et al*	2022	★	★	★	★	★	★	☆	☆	☆	7
Karpathiou G, *et al*	2022	★	★	★	★	★	★	☆	☆	☆	6
Zhan Z, *et al*	2023	★	★	★	★	★	★	★	★	★	9
Hyytiäinen A, *et al*	2023	★	★	★	★	★	☆	★	★	★	8

☆: scored 0, ★: scored 1.

Table S2. Association between positive-HEVs and clinicopathological parameters

Clinicopathological parameters	No.of studies	No. of patients	Effect model	Pooled OR (95%CI)	***P***	Heterogeneity I^2(%)	Heterogeneity ***P***	Relationship with positive-HEVs
Sex (Male vs. Female)	6	1192	Random	0.86, (0.50,1.49)	0.58	69	< 0.01	No statistical significance
Histologic grade (III vs. I-II)	4	418	fixed	1.74 (1.11, 2.73)	0.01	48	0.12	Positive
LVI (positive vs. negative)	3	237	fixed	1.05 (0.60, 1.86)	0.85	13	0.32	No statistical significance
Clinical stage (III-IV vs. I-II)	3	829	Random	0.86 (0.40, 1.85)	< 0.01	78	0.01	No statistical significance
Invasion of depth (I-II vs. III-IV)	6	1090	Random	0.91 (0.43,1.91)	0.80	83	0.01	No statistical significance
Lymph node status (N- vs N+)	6	1077	fixed	1.61 (1.22, 2.11)	< 0.01	34	0.18	Positive
Distant metastasis (M0 vs. M1)	3	362	fixed	~~3.32~~ (1.27, 7.87)	0.01	0	0.58	Positive

Abbreviation: LVI lymphovascular invasion, OR odds ratio

Figure S1.

A

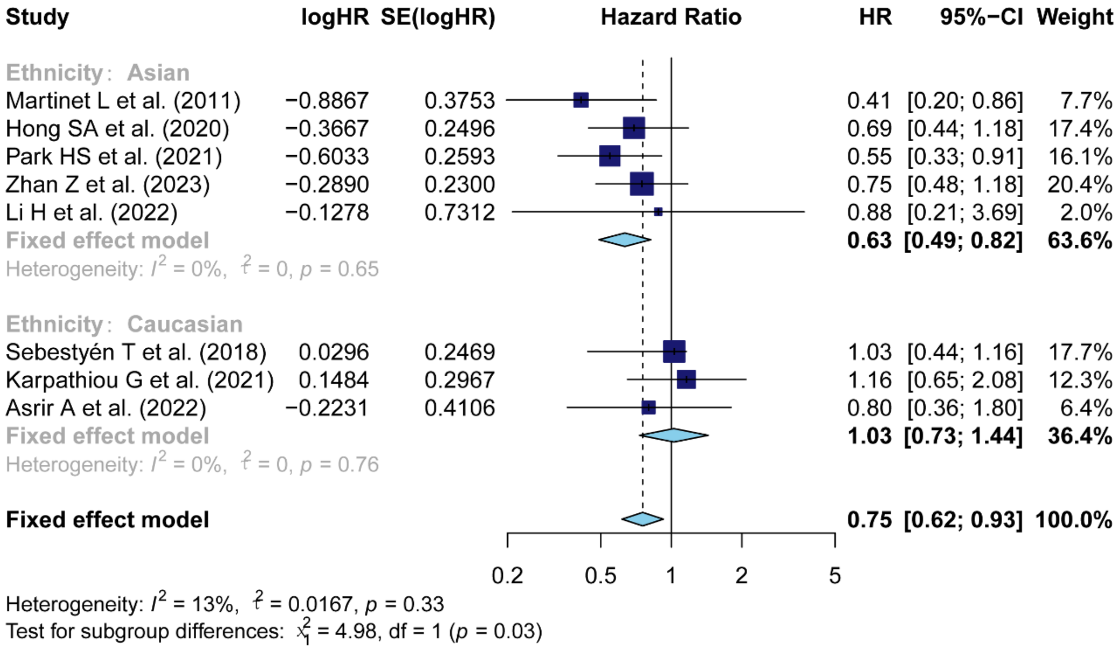
Study logHR SE(logHR) Hazard Ratio HR 95%−CI Weight
Ethnicity: Asian
Martinet L et al. (2011) −0.8867 0.3753 0.41 [0.20; 0.86] 7.7%
Hong SA et al. (2020) −0.3667 0.2496 0.69 [0.44; 1.18] 17.4%
Park HS et al. (2021) −0.6033 0.2593 0.55 [0.33; 0.91] 16.1%
Zhan Z et al. (2023) −0.2890 0.2300 0.75 [0.48; 1.18] 20.4%
Li H et al. (2022) −0.1278 0.7312 0.88 [0.21; 3.69] 2.0%
Fixed effect model 0.63 [0.49; 0.82] 63.6%
Heterogeneity: I^2 = 0%, τ^2 = 0, p = 0.65
Ethnicity: Caucasian
Sebestyén T et al. (2018) 0.0296 0.2469 1.03 [0.44; 1.16] 17.7%
Karpathiou G et al. (2021) 0.1484 0.2967 1.16 [0.65; 2.08] 12.3%
Asrir A et al. (2022) −0.2231 0.4106 0.80 [0.36; 1.80] 6.4%
Fixed effect model 1.03 [0.73; 1.44] 36.4%
Heterogeneity: I^2 = 0%, τ^2 = 0, p = 0.76
Fixed effect model 0.75 [0.62; 0.93] 100.0%
0.2 0.5 1 2 5
Heterogeneity: I^2 = 13%, τ^2 = 0.0167, p = 0.33
Test for subgroup differences: χ^2_1 = 4.98, df = 1 (p = 0.03)

B

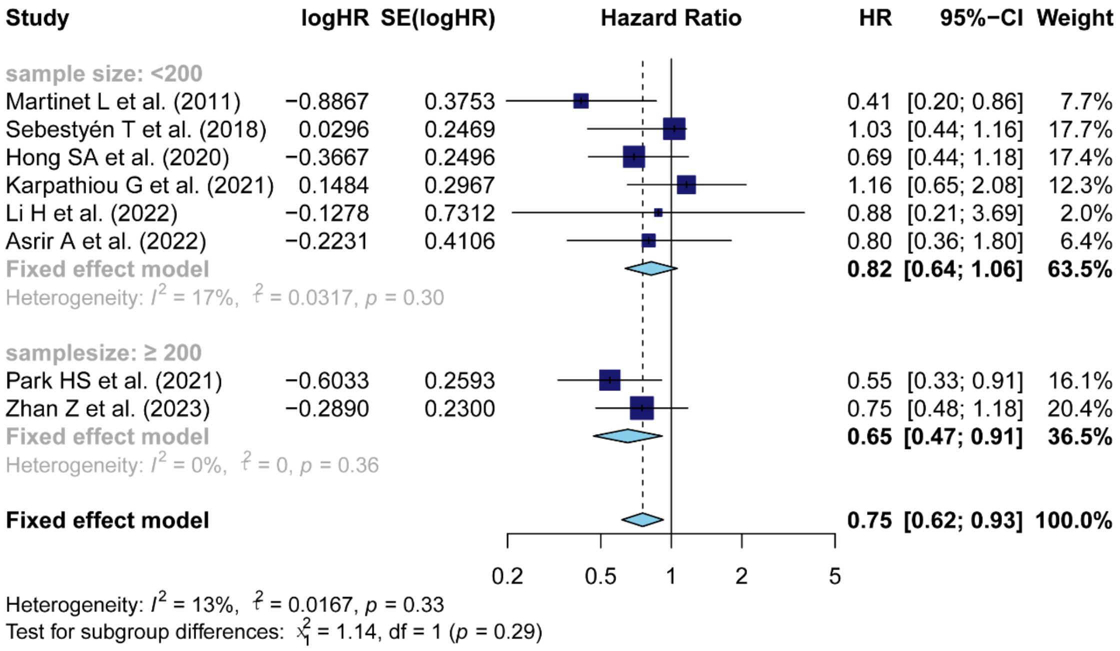
Study logHR SE(logHR) Hazard Ratio HR 95%−CI Weight
sample size: <200
Martinet L et al. (2011) −0.8867 0.3753 0.41 [0.20; 0.86] 7.7%
Sebestyén T et al. (2018) 0.0296 0.2469 1.03 [0.44; 1.16] 17.7%
Hong SA et al. (2020) −0.3667 0.2496 0.69 [0.44; 1.18] 17.4%
Karpathiou G et al. (2021) 0.1484 0.2967 1.16 [0.65; 2.08] 12.3%
Li H et al. (2022) −0.1278 0.7312 0.88 [0.21; 3.69] 2.0%
Asrir A et al. (2022) −0.2231 0.4106 0.80 [0.36; 1.80] 6.4%
Fixed effect model 0.82 [0.64; 1.06] 63.5%
Heterogeneity: I^2 = 17%, τ^2 = 0.0317, p = 0.30
samplesize: ≥ 200
Park HS et al. (2021) −0.6033 0.2593 0.55 [0.33; 0.91] 16.1%
Zhan Z et al. (2023) −0.2890 0.2300 0.75 [0.48; 1.18] 20.4%
Fixed effect model 0.65 [0.47; 0.91] 36.5%
Heterogeneity: I^2 = 0%, τ^2 = 0, p = 0.36
Fixed effect model 0.75 [0.62; 0.93] 100.0%
0.2 0.5 1 2 5
Heterogeneity: I^2 = 13%, τ^2 = 0.0167, p = 0.33
Test for subgroup differences: χ^2_1 = 1.14, df = 1 (p = 0.29)

C

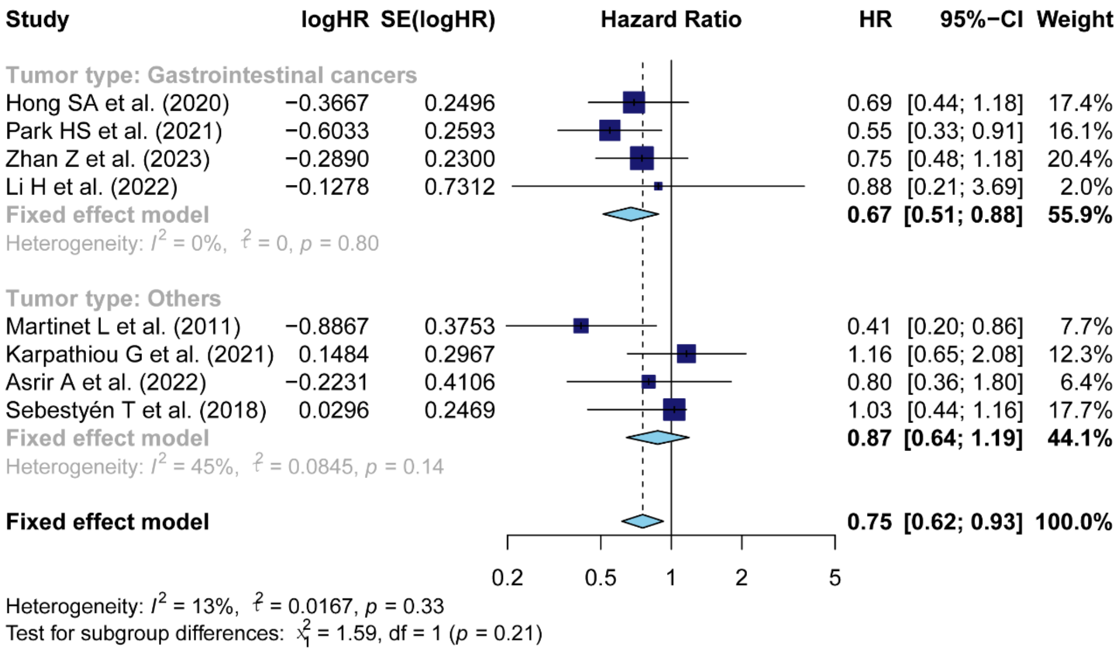

Study
logHR SE(logHR)
Hazard Ratio
HR 95%−CI Weight
Tumor type: Gastrointestinal cancers
Hong SA et al. (2020) −0.3667 0.2496 0.69 [0.44; 1.18] 17.4%
Park HS et al. (2021) −0.6033 0.2593 0.55 [0.33; 0.91] 16.1%
Zhan Z et al. (2023) −0.2890 0.2300 0.75 [0.48; 1.18] 20.4%
Li H et al. (2022) −0.1278 0.7312 0.88 [0.21; 3.69] 2.0%
Fixed effect model 0.67 [0.51; 0.88] 55.9%
Heterogeneity: I^2 = 0%, τ^2 = 0, p = 0.80
Tumor type: Others
Martinet L et al. (2011) −0.8867 0.3753 0.41 [0.20; 0.86] 7.7%
Karpathiou G et al. (2021) 0.1484 0.2967 1.16 [0.65; 2.08] 12.3%
Asrir A et al. (2022) −0.2231 0.4106 0.80 [0.36; 1.80] 6.4%
Sebestyén T et al. (2018) 0.0296 0.2469 1.03 [0.44; 1.16] 17.7%
Fixed effect model 0.87 [0.64; 1.19] 44.1%
Heterogeneity: I^2 = 45%, τ^2 = 0.0845, p = 0.14
Fixed effect model 0.75 [0.62; 0.93] 100.0%
0.2 0.5 1 2 5
Heterogeneity: I^2 = 13%, τ^2 = 0.0167, p = 0.33
Test for subgroup differences: χ^2_1 = 1.59, df = 1 (p = 0.21)

D

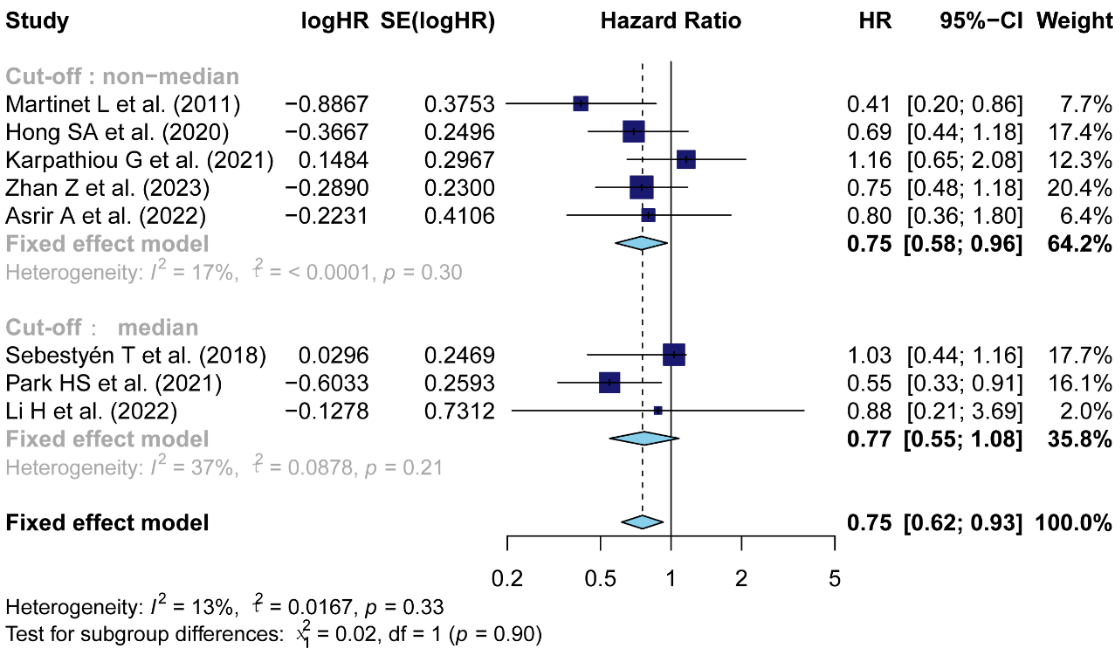

Study
logHR SE(logHR)
Hazard Ratio
HR 95%−CI Weight
Cut-off : non−median
Martinet L et al. (2011) −0.8867 0.3753 0.41 [0.20; 0.86] 7.7%
Hong SA et al. (2020) −0.3667 0.2496 0.69 [0.44; 1.18] 17.4%
Karpathiou G et al. (2021) 0.1484 0.2967 1.16 [0.65; 2.08] 12.3%
Zhan Z et al. (2023) −0.2890 0.2300 0.75 [0.48; 1.18] 20.4%
Asrir A et al. (2022) −0.2231 0.4106 0.80 [0.36; 1.80] 6.4%
Fixed effect model 0.75 [0.58; 0.96] 64.2%
Heterogeneity: I^2 = 17%, τ^2 = < 0.0001, p = 0.30
Cut-off : median
Sebestyén T et al. (2018) 0.0296 0.2469 1.03 [0.44; 1.16] 17.7%
Park HS et al. (2021) −0.6033 0.2593 0.55 [0.33; 0.91] 16.1%
Li H et al. (2022) −0.1278 0.7312 0.88 [0.21; 3.69] 2.0%
Fixed effect model 0.77 [0.55; 1.08] 35.8%
Heterogeneity: I^2 = 37%, τ^2 = 0.0878, p = 0.21
Fixed effect model 0.75 [0.62; 0.93] 100.0%
0.2 0.5 1 2 5
Heterogeneity: I^2 = 13%, τ^2 = 0.0167, p = 0.33
Test for subgroup differences: χ^2_1 = 0.02, df = 1 (p = 0.90)

E

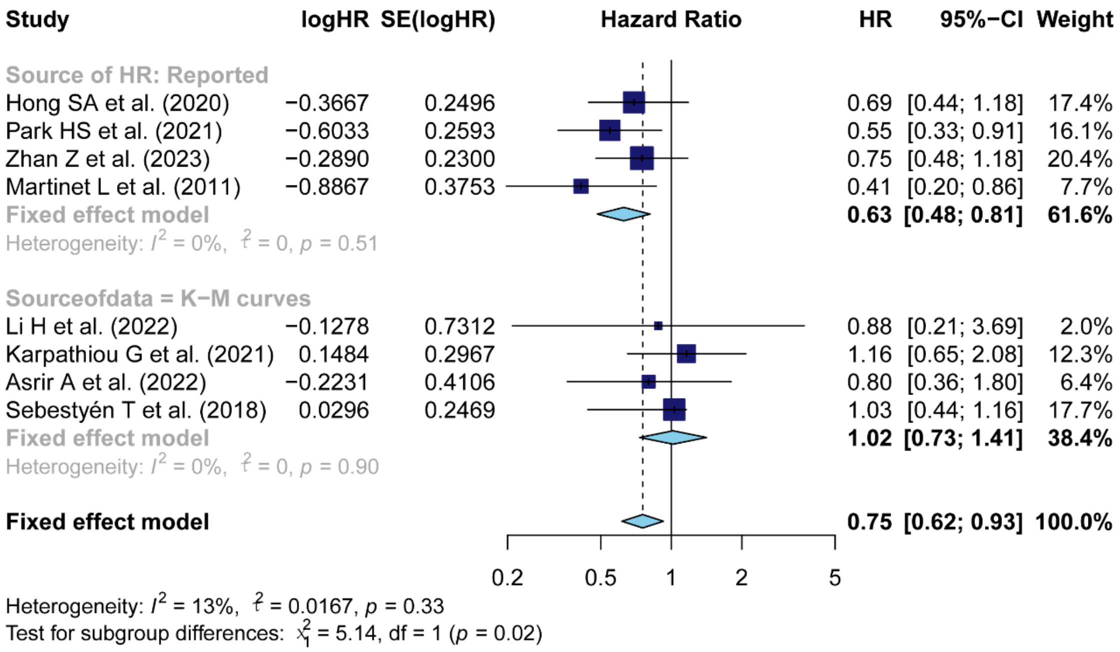

Figure S1. Subgroup analysis conducted for survival outcome regarding OS, stratified by (A) ethnicity, (B) sample size, (C) tumor type, (D) cut-off criteria, and (E) source of HR
